# Seroprevalence of *Toxoplasma gondii* infection among women of childbearing age in an endemic region of Romania, 2016–2018

**DOI:** 10.1051/parasite/2020057

**Published:** 2020-11-16

**Authors:** Alin Gabriel Mihu, Cornel Balta, Daniela Teodora Marti, Ana Alexandra Paduraru, Maria Alina Lupu, Tudor Rares Olariu

**Affiliations:** 1 Discipline of Parasitology, Victor Babes University of Medicine and Pharmacy Piata Eftimie Murgu, No. 1 300041 Timisoara Romania; 2 Bioclinica Dreptatii Street, No. 23, Bl. 707 310300 Arad Romania; 3 Vasile Goldis Western University Liviu Rebreanu Street, No. 86 310048 Arad Romania; 4 Clinical Laboratory, Municipal Clinical Emergency Hospital Strada Gheorghe Dima Nr. 5 300254 Timisoara Romania; 5 Clinical Laboratory, Institute of Cardiovascular Diseases Strada Gheorghe Adam Numarul 13A 300310 Timisoara Romania; 6 Center for Diagnosis and Study of Parasitic Diseases, Victor Babes University of Medicine and Pharmacy Piata Eftimie Murgu, No. 1 300041 Timisoara Romania

**Keywords:** Toxoplasmosis, Women of childbearing age, Laboratory diagnosis, Seroprevalence

## Abstract

Toxoplasmosis is an important worldwide zoonosis caused by the protozoan parasite *Toxoplasma gondii*. This parasitic infection is often asymptomatic in immunocompetent people. However, if the infection occurs in pregnant women, it can have serious consequences for the foetus. In this study, we evaluated the seroprevalence of *T. gondii* in women of childbearing age in Arad County, Western Romania. Serum samples from 2626 women were analysed using a Siemens ADVIA Centaur XP Immunoassay System. *Toxoplasma gondii* IgG antibodies were demonstrated in 1081 women (41%) and prevalence tended to increase with age, from 32% in women aged 15–19 years to 62% in women aged 40–45 years. There was a higher prevalence in rural areas (46%) than in urban areas (36%). This study provides new data on *T. gondii* seroprevalence in women of childbearing age from Western Romania.

## Abstract (fr)

La toxoplasmose est une zoonose mondiale importante causée par le parasite protozoaire *Toxoplasma gondii*. Cette infection parasitaire est souvent asymptomatique chez les sujets immunocompétents. Cependant, si l’infection survient chez la femme enceinte, elle peut avoir de graves conséquences pour le fœtus. Dans cette étude, nous avons évalué la séroprévalence de *T. gondii* chez les femmes en âge de procréer dans le comté d’Arad, dans l’ouest de la Roumanie. Des échantillons de sérum de 2626 femmes ont été analysés avec le Siemens ADVIA Centaur XP. Des anticorps IgG contre *T. gondii* ont été mis en évidence chez 1081 femmes (41 %) et la prévalence avait tendance à augmenter avec l’âge, passant de 32 % chez les femmes âgées de 15 à 19 ans à 62 % chez les femmes âgées de 40 à 45 ans. La prévalence était plus élevée dans les zones rurales (46 %) que dans les zones urbaines (36 %). Cette étude fournit de nouvelles données sur la séroprévalence de *T. gondii* chez les femmes en âge de procréer de l’ouest de la Roumanie.

## Introduction

*Toxoplasma gondii* is a single-celled, obligate intracellular protozoan parasite. Contact with this parasite causes the infection called toxoplasmosis. In humans, the infection is transmitted either by ingestion of food or water contaminated with oocysts or by eating undercooked or raw meat that contains tissue cysts [13]. Usually, in immunocompetent individuals, toxoplasmosis remains asymptomatic and undiagnosed. If *T. gondii* primary infection occurs during pregnancy, this parasite may cause congenital infection with devastating consequences to the infected child, including chorioretinitis, blindness, cerebral calcifications, hydrocephalus, microcephaly, or developmental delay. Due to the severe complications of congenital toxoplasmosis, the seroprevalence of *T. gondii* in women of childbearing age should be monitored [2, 16, 18].

Seroprevalence of *T. gondii* IgG antibodies in women of childbearing age varies between countries and sometimes between regions within a country, ranging from 4% in South Korea to 84% in Madagascar [6]. A higher rate of latent toxoplasmosis has been associated with low-income, developing countries [23]. Developing countries in Africa report a high prevalence. In Ethiopia, for example, seroprevalence of *T. gondii* IgG antibodies in women of childbearing age is as high as 78.4% [8], and in Cameroon the seroprevalence of these antibodies is 54% [26]. In highly industrialised countries such as the United States, seroprevalence of *T. gondii* IgG antibodies is around 9% [10].

The overall seroprevalence of *T. gondii* IgG antibodies in Europe, in women of childbearing age, was estimated at 23% in 2014 [6]. Seroprevalence varies in different countries: 37% in France [14], 25.9% in Germany [27], 33% in Serbia [22], and 29.1% in Croatia [25].

In Romania, there is a lack of knowledge on the current epidemiological situation regarding toxoplasmosis, because only small-scale studies have been performed until now. In addition, no national screening programme for pregnant women and congenital toxoplasmosis have been implemented. Previous reports have shown that *T. gondii* infection is endemic in Western Romania [15, 17]. However, no studies regarding *T. gondii* seroprevalence have been conducted among women of childbearing age in Arad County, Western Romania. Therefore, we decided to assess the seroprevalence of *T. gondii* infection in women of childbearing age from this region.

## Materials and methods

We investigated serum samples collected from 2626 women of childbearing age. The women were 15–45 years of age and were residents of Arad County, which has a population of 409,072 inhabitants. Samples were collected from 01 January 2016 to 31 December 2018, and no clinical criteria were used to include individuals in this study.

Women were grouped into six categories based on their age when the sample was drawn: 15–19 years, 20–24 years, 25–29 years, 30–34 years, 35–39 years, and 40–45 years.

Serum samples were screened for IgG anti-*T. gondii* antibodies using ADVIA Centaur^*®*^ XP (Siemens Healthcare Diagnostics, USA), according to the manufacturer’s instructions and internal laboratory standards. This test detects IgG antibodies using a chemiluminescence method. A value above 10 IU/mL was considered positive, inconclusive values ranged between 6.4 and 10 IU/mL, and values below 6.4 IU/mL were negative. For the purposes of this study, inconclusive values were considered negative.

Data were collected using Microsoft Excel, version 2011 (Microsoft Corp., Redmond, WA, USA), and statistical analyses were performed with the Epi Info statistical package 3.3.2 (Centers for Disease Control and Prevention, Atlanta, GA, USA). Mantel–Haenszel chi-square and two-tailed Fisher’s exact tests were used for comparison between groups. A *p*-value of <0.05 was considered statistically significant.

This study was approved by the Victor Babes University Ethics Committee, Timisoara, Romania.

## Results

*Toxoplasma gondii* IgG antibodies were found in 1081 of 2626 females (41.16%), and the prevalence tended to increase with age. A statistically significant higher seroprevalence was observed in women 40–45 years old (61.53%) when compared to those 15–19 years (32.14%) (*p* = 0.03). No statistically significant difference in seroprevalence was found when we compared women 15–19 years (32.14%) with those 20–24 years (38.92%) (*p* = 0.28), 24–29 years (42.79%) (*p* = 0.18), 30–34 years (39.57%) (*p* = 0.27), and 35–39 years (39.42%) (*p* = 0.28) (Table 1). When data were analysed according to the area of residence (urban or rural) a higher seroprevalence of IgG antibodies was found in women from rural areas (46.06%, 614/1333) when compared to women from urban areas (36.11%, 467/1293) (*p* = 0.001) (Table 1).

**Table 1:** Seroprevalence of *Toxoplasma gondii* infection in women of childbearing age according to age and area of residence.

Age (years)	No. positive/No. investigated (%)			*p*-values
	Urban	Rural	Overall	
15–19	4/18 (22.22%)	14/38 (36.84%)	18/56 (32.14%)	–
20–24	49/128 (38.28%)	111/283 (39.22%)	160/411 (38.92%)	0.28
25–29	155/442 (35.06%)	270/551 (49.00%)	425/993 (42.79%)	0.18
30–34	148/428 (34.57%)	131/277 (47.29%)	279/705 (39.57%)	0.27
35–39	83/235 (35.31%)	68/148 (45.94%)	151/383 (39.42%)	0.28
40–45	28/42 (66.66%)	20/36 (55.55%)	48/78 (61.53%)	0.03
Total	467/1293 (36.11%)	614/1333 (46.06%)	1081/2626 (41.16%)	–

The *T. gondii* IgG antibodies seroprevalence tended to increase with age in both rural and urban areas.

## Discussion

This study demonstrated the presence of *T. gondii* IgG antibodies in serum samples of 41.16% of women of childbearing age living in Arad county, a well-known endemic area of Romania. The seroprevalence was higher than that reported by other European investigators: 24.1% in Northern Kosovo and Metohija [22], 22.3% in Italy [5], 22% in Portugal [7], and 29.1% in Croatia [25]. The differences in seroprevalence of *T. gondii* IgG antibodies between countries can be explained by different nutritional habits, as well as sociodemographic and cultural factors [17, 19]. However, seroprevalence of *T. gondii* infection in our study was lower than the 57.6% reported in women of childbearing age from Timis county, Romania, one of the neighbouring counties [15]. *Toxoplasma gondii* seroprevalence may vary within a country and sometimes between different communities in one region [17, 20]. We have no explanation for this difference because participants were not interviewed regarding additional risk factors such as meat consumption, contact with cats or soil, occupation, economic status, or education level. It is possible that some of these potential exposure factors could contribute to the difference in *T. gondii* seroprevalence between Arad and Timis counties.

In the current study, the seroprevalence was higher in women from rural areas (46.06%) compared to those from urban areas (36.11%). Similar findings were reported by Olariu et al. and Vilibic-Cavlek et al. [15, 25]. This can be explained by the activities carried out in rural areas which may expose individuals to contaminated water or soil [24]. Farming, gardening, and handling animals are factors that may contribute to a higher prevalence in women residing in rural areas [12]. Prevalence rates are influenced by geographic differences and may be explained by exposure to the main sources of infection: tissue cysts acquired from undercooked meat, or oocysts found in soil contaminated by cat faeces. Feral cats that defecate in gardens or sandboxes may pose a risk of *T. gondii* infection in some people, regardless of whether they own a cat [1, 3, 9, 11].

As previously reported by other authors, seroprevalence of *T. gondii* infection in our study showed an age-related increase, with lower rates found in younger women. This type of association probably occurred due to longer exposure to the risk factors associated with *T. gondii* infection [19, 21].

No epidemiological investigations or questionnaires were conducted in this study. However, a seroprevalence of 70% of *T. gondii* antibodies was previously reported in cats in Western Romania, which may contribute to the high seroprevalence in our studied group [4].

The present study brings new epidemiological information on the prevalence of *T. gondii* infection in women of childbearing age from Arad County, Western Romania. Our results indicate a high prevalence of *T. gondii* infection in this study group. Further studies should be conducted to determine the main risk factors that contribute to such seroprevalence. This knowledge is vital to predict and prevent the risk of infection in pregnant women in the future. The serological status of women of childbearing age provides valuable information on immunity, which may help to prevent congenital infection by identifying women at risk. Our findings may serve as a starting point for counselling and education programmes on toxoplasmosis in antenatal clinics, and for the implementation at the national level of a screening and prevention programme for pregnant women.

## Declaration of interest

The authors report that they have no conflicts of interest. The authors alone are responsible for the content and writing of the paper.
